# Spastic paraplegia is the main manifestation of a spinocerebellar ataxia type 8 lineage in China: a case report and review of literature

**DOI:** 10.3389/fnhum.2023.1198309

**Published:** 2023-07-17

**Authors:** Shuling Chen, Siyu Li, Ying Liu, Renyi She, Wei Jiang

**Affiliations:** Department of Rehabilitation, Children's Hospital of Chongqing Medical University, Chongqing, China

**Keywords:** spinocerebellar ataxia type 8, spastic paraplegia, cerebellar atrophy, antispasmodic rehabilitation, case report

## Abstract

The diagnosis and treatment of cerebellar atrophy remain challenging owing to its nonspecific symptoms and laboratory indicators. Three patients with spinocerebellar ataxia type 8 caused by *ATXN8OS* were found among the 16 people in the studied family. The clinical manifestations of the patients included progressive spastic paraplegia of the lower extremities, mild ataxia, mild cognitive impairment, and cerebellar atrophy. After administering antispasmodic rehabilitation treatment, using oral drugs, botulinum toxin injection, baclofen pump, and other systems in our hospital, the patients' lower extremity spasticity was significantly relieved. To our knowledge, till date, this is the first domestic report of spinocerebellar ataxia type 8 affecting a family, caused by *ATXN8OS* with spasticity onset in early childhood. Manifestations of the disease included spastic dyskinesia (in early disease stages) and cerebellar atrophy. Through systematic rehabilitation, the daily life of patients with this movement disorder was improved. This case report adds to the literature on spinocerebellar ataxia type 8 by summarizing its features.

## Introduction

Spinocerebellar ataxia (SCA) is an autosomal dominant neurodegenerative disease that occurs mostly in adults, with progressive ataxia as the main manifestation often accompanied by dysarthria, dystonia, and oculomotor nerve control disorders (Wagner et al., 2016; Aydin et al., 2018; Buijsen et al., 2019). The main pathological changes involve the neural tissue in the cerebellar dentate nucleus, brainstem, basal ganglia, and substantia nigra, where nerve atrophy is the dominant feature (Koeppen, 2018). SCA occurs worldwide, with an overall prevalence ranging from 0.3 to 4.2 cases per 100,000 people (Erichsen et al., 2009). Among SCA variants, SCA type 8 (SCA8) is related to simple cerebellar ataxia, and its pathogenic gene is the 3′ untranslated region of chromosome 13q21 (*ATXN8OS* in humans) (Koob et al., 1999). SCA8 CTG repeat amplification may play a role in the development of sporadic or atypical Parkinson's diseases (Wu et al., 2004). In addition to this amplification occurring in *ATXN8OS*, SCA8 involves it occurring in another overlapping gene, ataxin 8 (*ATXN8)* (Ikeda et al., 2008). In the CTG direction, *ATXN8OS* expresses a non-encoded transcript containing CUG amplification, which overlaps the 5′ region of the Kelch-like 1 (KLHL1) transcript. In the CAG direction, *ATXN8* expresses a transcript encoding the polyglutamine amplified protein (Ayhan et al., 2018). Thus, three possible mechanisms could be responsible for the pathophysiology of SCA8, RNA function acquisition, partial loss of KLHL1 function, and CAG-oriented polyglutamine amplification protein (Ranum and Day, 2004; He et al., 2006; Moseley et al., 2006; Ayhan et al., 2018).

SCA8 was first reported in 1999 (Koob et al., 1999; Cintra et al., 2017). Its clinical manifestations are gait, limb, speech, and oculomotor nerve incoordination; spasm; and sensory impairment (Mosemiller et al., 2003). Additionally, patients show cognitive impairment, epilepsy, and psychiatric symptoms (Lilja et al., 2005; Torrens et al., 2008; Swaminathan, 2019). Furthermore, cerebellar atrophy may be revealed from imaging examination.

We analyzed SCA8 by reporting the clinical data, auxiliary examinations, genetic test results, and systematic rehabilitation treatment process of the ATXN8OS-SCA8 family and reviewed the relevant literature.

## Case report

**Figure 1:**
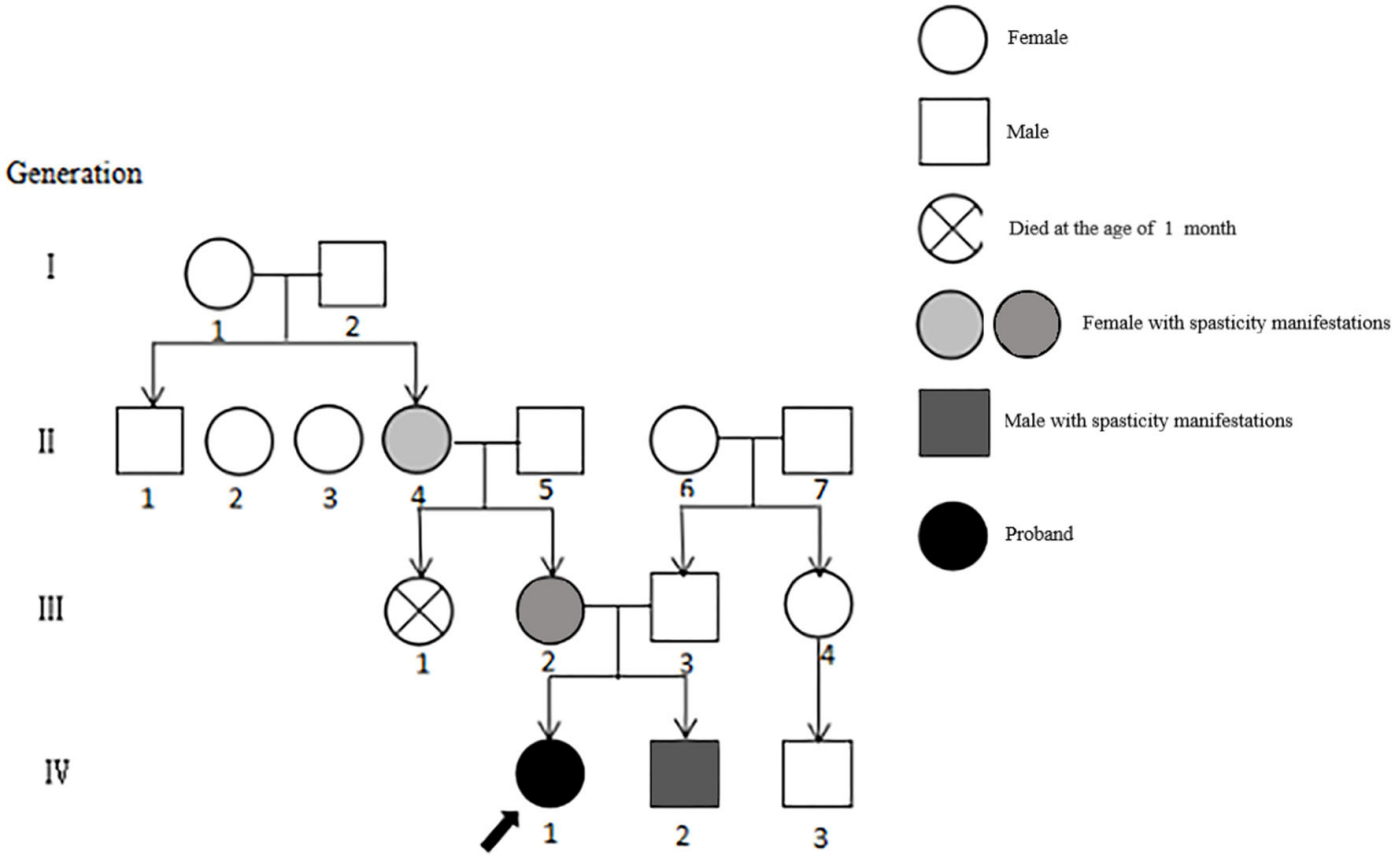
Genealogy of 4 generations of patients with SCA8. The severity of the patient's symptoms is indicated by light and dark colors, and the order is II4, III2, IV2, and IV1.

The patient (Figure 1) was a girl aged 10 years and 1 month. She was more than 9 years behind in normal motor development. The disease was initially identified 9 months after birth because she displayed stagnation in motor development such as sitting unsteadily on her own, increased muscle tone in the lower limbs, and mild dysarthria. Her condition gradually worsened even with intermittent rehabilitation. Presently, she can only walk with a tractor in both hands, displaying pathologically pointed feet, knee flexion, and hip flexion gait. The patient exhibits mild nystagmus, an inability to bend down, sit forward, sit up straight, pull objects, and stand on her own. She has an abnormal finger-nose test result, intentional tremor, V grade limb muscle strength, Achilles tendon contracture, internal foot rotation (varus), horseshoe foot, bicep, knee tendon reflex, hyperreflexia, and a bilateral positive Babinski sign. The clinical data of the proband and her family are shown in Table 1.

**Table 1:** Clinical data of the progenitors and her families.

**Clinical features**	**People with symptoms in the family**				**Asymptomatic people in the family lineage**
	**Proband (IV1)**	**Brother (IV2)**	**Mom (III2)**	**Grandmother (II4)**	**Others**
Age	10 y	6 y	32 y	63 y	/
Sex	Female	Man	Female	Female	/
Age of onset	9 m	1 y	Unknown	60 y	—
Primary symptoms	Moderate paraplegia gait	Mild paraplegic gait	Pointed feet	Pointed feet	—
SARA rating	64111122	22111111	10000100	/	—
Unstable gait	Moderate	Mild	—	Mild	—
Finger-nose test	+	+	—	—	—
Romber	+	+	—	—	—
Nystagmus	+	—	—	—	—
Dysarthria	+	+	—	—	—
Intellect	Edge	Edge	—	—	—
MAS rating	2-3 levels	1-1+levels	1 levels	/	—
Spastic posture	Moderate	Mild	—	—	—
Hyperreflexia	+	+	—	/	—
Babinski sign	+	+	—	/	—
Ankle reflex	+	+	—	/	—
MRI	Cerebellar atrophy	Cerebellar atrophy	—	/	—

MAS, Modified Ashworth Scale; MRI, magnetic resonance imaging; SARA, scale for the assessment and rating of ataxia.

**Figure 2:**
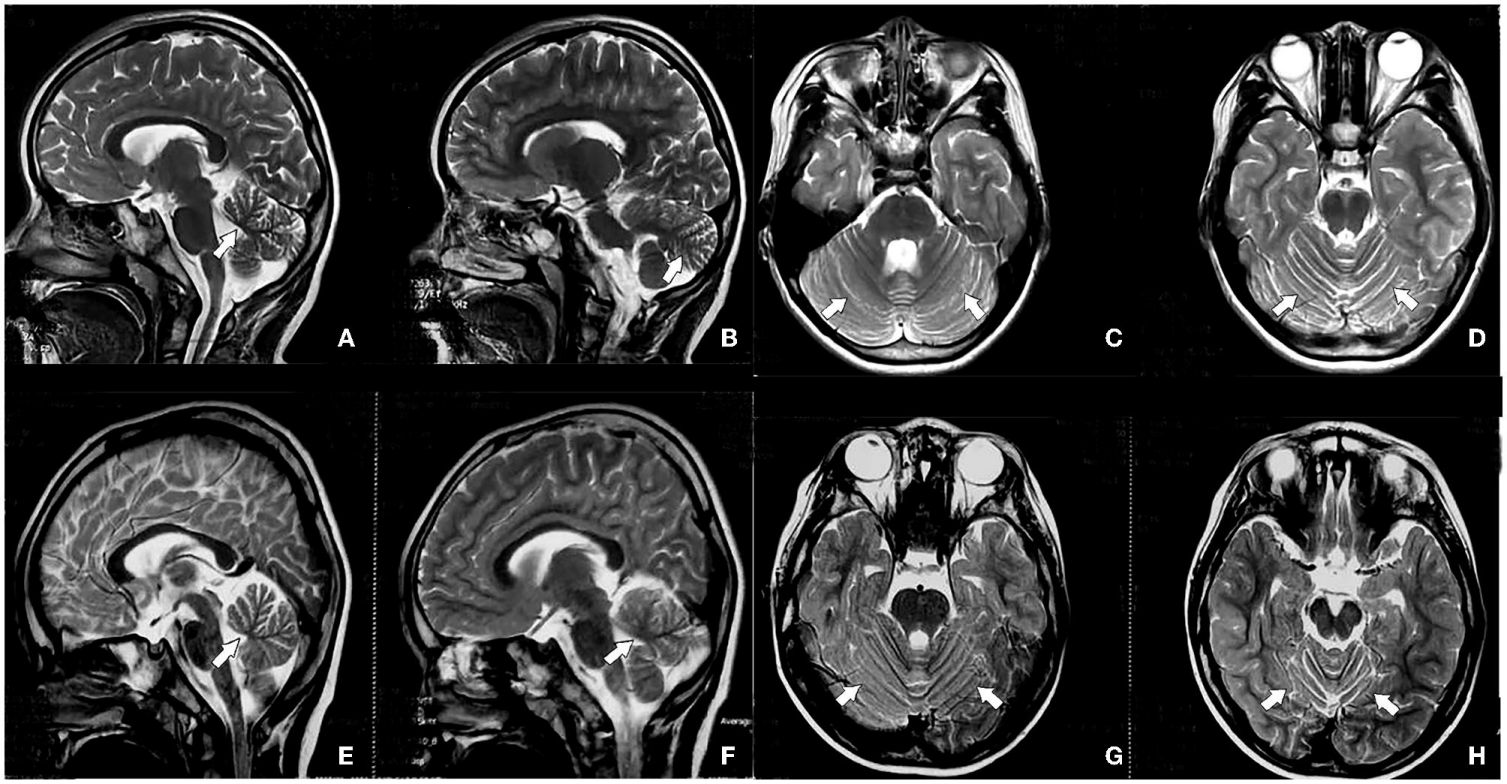
Head magnetic resonance imaging (MRI) of the progenitor (IV.1) shows cerebellar atrophy **(A–D)**, and head MRI of the brother of the predominant (IV.2) shows cerebellar atrophy **(E–H)**, indicated by the white arrow.

Routine blood, liver and kidney function, electrolytes, and myocardial and muscle enzyme profile test results were normal. Urinalysis suggested that the qualitative urinary calcium test++ was higher than normal. Magnetic resonance imaging (MRI) suggested no abnormality in the panspinal spondyloma of the progenitor, but cerebellar atrophy was seen in a flat skull scan (Figures 2A–D). Double lower limb joint muscle ultrasound showed (1) bilateral flounder muscle and hamstring muscle echo enhancement, muscle fiber loss disorder, and a vague feather structure; and (2) bilateral gastrocnemius acoustic imaging with no obvious abnormalities. X-ray foot imaging was suggestive of osteoporosis of the right foot and varus of the right foot with an enlarged arch. Electroencephalography (EEG) findings were normal. The Gross motor function measure-88 and Fine motor function measure showed that the patient's current gross and fine motor skill development was behind that of children of the same age. The Wechsler test results were as follows: language test intelligence quotient (IQ) = 82, operational test IQ = 78, and total IQ = 77 (indicative of critically low intelligence).

The second-generation total exon sequencing technique was used to analyze the ataxia caused by single-gene mutations; additionally, the dynamic mutations and repetitions were detected by using capillary electrophoresis sequencing technology to verify and analyze the mutation sites of the patient and her family members. The results of the pre-existent (IV.1) gene tests of her and her brother showed variation in *ATXN8OS*. The capillary electrophoresis method suggested that the number of CTG triple nucleotide repeats in *ATXN8OS* coding area was abnormal, totaling >400 times (SCA8 diagnostic reference value: normal repetitions = 15–50; abnormal repetitions = 70–1300) (Koob et al., 1999). Owing to limitations of the genetic company's testing equipment, capillary electrophoresis did not have a cliff-like interruption, and the specific number of replicates could not be detected. The number of CTG triple nucleotide repeats in the CTG coding region of *ATXN8OS* was >300 times for her mother. Based on these results, the genotype of the family was confirmed as SCA8.

The proband received antispasmodic rehabilitation. According to her clinical performance, the first stage of systematic physical therapy was selected. She was guided by a professional rehabilitation therapist in our department. Her program included physiotherapy training (2 times a day for 50 min), gait training (treadmill) (once a day for 30 min), and instrument training using passive traction instruments (20 min/group, 10 times/group, 6 groups/day). In the case of poor physical system rehabilitation, the second stage was supplemented with a knee and ankle-foot orthosis, oral clonazepam (0.05 mg/kg/d, bid; later adjusted to 0.1 mg/kg/d, bid), oral baclofen (0.5 mg/kg/d, tid; later adjusted to 1 mg/kg/d, tid), and botulinum toxin injection for almost 1 year to improve the lower muscle tone (400 units, the injection sites were the bilateral hamstring, flounder, and gastrocnemius muscles; after 2 months, 200 units were added, the injection sites were the bilateral iliopsoas and adductor muscles). The third phase involved the surgical implantation of a baclofen pump system (Medtronic SynchroMed II infusion system) in March 2022. It was placed between the third fourth lumbar vertebral bodies. The pump administered 140 μg of baclofen daily.

## Results

Sixteen patients from four generations of the patient's family lineage were examined. Her grandmother (II.4), mother (III.2), and brother (IV0.2) had similar manifestations to those of the proband (Figure 1). At 60 years, II.4 displayed mild increased muscle tone in the ankle muscle group, mild pointed feet, and occasional falls but did not regard herself as having obvious abnormalities. III.2 had a pointed foot and could not squat with flat feet; however, she displayed no head-related abnormalities upon MRI examination and was unhindered in her daily life. IV0.2, aged 6 years, displayed walking instability since he was 1 year old, mild spastic paraplegia of both lower limbs, paraplegic gait, mild ataxia with mild impairment in walking, no abnormalities on EEG, and cerebellar atrophy (Figures 2E–H). III.2 and IV.2 were genetically tested and diagnosed with SCA8. The same diagnosis was inferred for II.4, based on her history. All cases underwent total penetrance detection and copy number variation and indicated no genetic variants, such as hereditary spastic paraplegia.

Before admission to our hospital, the proband's modified Rankin Scale (mRS) score was level 4, and the Modified Ashworth Scale (MAS) score of her lower limb flexor was level 3. A reduction of mRS or MAS level by 1 is considered a moderate treatment response, while a reduction by 2 is considered a marked treatment response (Gomez-Cuaresma et al., 2021). During her first stage of treatment in our hospital, we began exercise training, gait training, and instrument training, but the effect on the patient was negligible. In the second stage of treatment, the patient was given additional knee and ankle-foot orthoses, oral baclofen, and clonazepam for almost 1 year to improve her lower muscle tone. As a result, her MAS grade decreased by nearly 1 level, but she still showed a significant paraplegic gait and could not stand on her own. The addition of botulinum toxin injection still showed no improvements. The third stage of treatment entailed surgical implantation of a baclofen pump in the patient. The patient could now stand on her own, with slight hip and knee flexion, walk on flat ground with aid, and showed an improved mRS score and MAS score of her lower limb flexors (see Table 2). Her brother also underwent sports training, gait training, and equipment training in our hospital. Ankle-foot orthoses were also installed since these are currently more effective in the treatment of mild paraplegia gait. The brother is now able to attend school freely.

**Table 2:** Follow-up of rehabilitation effects of proband.

	**modified Rankin Scale (proband)**	**MAS (proband)**
Before coming to the hospital	Level 4	Level 3
Athletic training Gait training Apparatus training	Recovery is less effective	
The first stage of treatment is completed	Level 4	Level 2-3
Oral hypotonia medications (Clonazepam, baclofen) Knee and ankle orthoses Botulinum toxin injection	Pronounced paraplegic gait can't stand alone and walk away	
The second stage f treatment is completed	Level 4	Level 2
Baclofen pump	Stand alone, squat a little walk flat with other's help	
The third stage of treatment is completed	Level 3	Level 1+

MAS, Modified Ashworth Scale. Adjustment of the treatment plan duration was based on the outcome of the prior treatment and assessment of the effectiveness of treatment using mRS and MAS ratings.

## Discussion

SCA is a neurogenetic degenerative disease with many pathogenic genes. Genetic testing is currently the most efficient means of diagnosis, and over 40 subtypes of SCA exist (Ruano et al., 2014; Wang et al., 2019). The prevalence of SCA subtypes varies according to geographical region. For example, SCA type 10 cases are concentrated in Mexico and Brazil (Teive, 2009; Teive et al., 2010), and the prevalence of SCA type 3 (SCA3) is the highest in China (Hao et al., 2020), where most cases affect adults (Zeigelboim et al., 2015; Zhou et al., 2019). According to data from our hospital, single nucleotide variation, with fewer dynamic repeating mutations, are responsible for the majority of SCA cases in children. SCA8 is a relatively uncommon form of slow-progressing ataxia, with an estimated global prevalence of 1 in 100,000 people, which varies by ethnicity and region (Recent, 2000; Cintra et al., 2017).

SCA8 is mainly associated with cerebellar ataxia and first appears between the ages of 18 and 65 years, with an average age of onset of 39 years (Hu et al., 2017), As SCA cases continue to accumulate, the symptom spectrum of SCA is expanding, from the classic manifestations of ataxia to Parkinson's, dementia, paroxysmal kinesigenic dyskinesia, epilepsy, cognitive impairment, and executive dysfunction (Torrens et al., 2008; Reetz et al., 2012; Swaminathan, 2019). Presently, we report the first family of SCA8 caused by *ATXN8OS* with spasticity paraplegia onset in early childhood in China (Wang et al., 2018; Guo et al., 2022). In the present case, both the proband and her brother were affected since childhood. SCA8 has a later onset time than SCA3, but their clinical manifestations are similar (Tao et al., 2021). Our patients' disorders were characterized by progressive spastic paraplegia of the lower extremities, mild ataxia, and a clear sign of vertebral bundle damage on physical examination.

The neuropathology of SCA8 is unclear now, and the studies have revealed several shared characteristics in the affected individuals, including pronounced neuronal loss in the substantia nigra, loss of Purkinje neurons, atrophy of the molecular layer, and heightened proliferation of radial glia in the cerebellum (Yonenobu et al., 2023). Furthermore, polyglutamine monoclonal antibody 1C2-positive intranuclear inclusions and pan-nuclear staining were observed in the Purkinje, medulla, and dentate nucleus of the human SCA8 brain. These observations are a result of translation of a polyglutamine protein (Moseley et al., 2006). However, children with SCA8 and their parents share pyramidal tract involvement in our report and a 10 years child (Zhou et al., 2019). This suggests that spasticity symptoms occurring owing to pyramidal tract injury in children with SCA8 may not be a rare clinical manifestation. The data obtained indicate that the clinical pathogenesis relies on the intranuclear accumulation of mutant proteins in neurons, resulting in cell degeneration (Moseley et al., 2006; Yamada et al., 2008; Martí, 2016); According to a study conducted by Gu et al., it was observed that polyalanine aggregation was present in the brain tissue of patients with SCA8 as well as in model mice. We can infer that this localized accumulation of toxic proteins could potentially lead to the impairment of motor cortex neurons and subsequently affect the pyramidal tract (Zhou et al., 2020). Presently, there are no reports indicating whether the lesion site of SCA differs for adult and pediatric patients, but since most adults with SCA are characterized by cerebellar ataxia, we can stipulate that patients with SCA with a late onset have cerebellar involvement, and those with an early onset have pyramidal involvement that can slowly involve the cerebellum and other parts (Yamada et al., 2008). However, the above are all conjectures, and relevant studies will need to be made in the future.

Degenerative changes and atrophy of the cerebellar nerves in patients with SCA8 are potential physiological causes of loss of motor coordination and speech difficulties—traits that mark the onset and progression of the disease (Mutsuddi and Rebay, 2005). The patients in this case also had mildly impaired cognition, with Wechsler test results suggesting critically low intelligence levels. The affective disorder is not obvious in this case, and epilepsy (although uncommon) has been reported in patients with SCA8 (Swaminathan, 2019). In our proband, no abnormal epileptic discharges were found on EEG. MRI scans of SCA8 show marked atrophy of the cerebellar hemisphere and mild atrophy of the brainstem. Cortical atrophy has been detected in only a few cases (Juvonen et al., 2000). Previous studies' MRI findings were consistent with those of our patients. Conventional structural MRI has become the standard care method for monitoring cerebellar and brainstem atrophy in patients with SCA (Ashizawa et al., 2018). Presently, electrophysiological research on neurodegenerative diseases is active, such as EMG, MEP, and SEP. Some studies have found that electrogastrography can diagnose Parkinson's disease early (Araki et al., 2021), and visual evoked potentials can assess the severity of Parkinson's disease (Garcia-Martin et al., 2014). Therefore, non-invasive tests can help diagnose, identify and evaluate the course of the disease. However, studies examining neurodegenerative diseases that affect pediatric patients are few, and the use of electrophysiology is insufficient for their diagnosis. As a result, the electrophysiological examination of this family case is not perfect. Subsequent studies should attempt to improve the relevant electrophysiological assessment.

Owing to the patient's young age and spastic paraplegia of both lower limbs being the main manifestation, the patient's family refused the routine histoneuropathological examination. However, her ataxia was obvious through physical examination, considering the nystagmus present. MRI of the skull revealed the following: decreased cerebellar volume, mainly the cerebellar vermis; cerebellar body sulcus deepening; and brain thinning, suggesting changes due to cerebellar cell apoptosis loss. *ATXN8OS* CTA or CTG repeat sequence amplification has also been reported in other neurodegenerative diseases (Sawada et al., 2020) and psychiatric disorders (Torrens et al., 2008). Through total exon sequencing, we found a variant of *ATXN8OS* in the proband, with the mother (III.2) and the proband's brother (IV.2) displaying the same mutation. Genetic test results showed that the CTA/CTG repeat sequence occurred >71 times, in line with the genetic mutation characteristics of SCA8. The patient's grandmother did not undergo genetic testing owing to external factors, but SCA was suspected because of her clinical presentation. Additionally, in this family, we found significant variations in the severity and penance of the disease. SCA8 is a rare, low-eminent, CTA/CTG repeat disease, and the expansion of CTA/CTG is highly unstable between generations (Paganoni et al., 2008; Buijsen et al., 2019). Paternal propagation usually causes CTG repeat contraction, while maternal propagation usually leads to CTG repeat dilation (Ikeda et al., 2004; Mutsuddi and Rebay, 2005). Clinical manifestations of SCA occurred in the grandmother, mother, proband, and the proband's brother; thus, maternal transmission was suspected, and the clinical dysfunction of the predeterminant siblings was predisposed. The number of nucleotide repetitions was dilated, which is consistent with the characteristics of maternal transmission. However, because of its genetic complexity, low penance, and clinical diversity, the effect of SCA8 on offspring is difficult to assess. In this case, the improvement of motor symptoms was obvious, and the self-before and after-comparison of individual cases were made. However, there is a lack of large-sample clinical prospective controlled studies in this area, so its effectiveness for most patients with SCA8 could not be determined. Therefore, the next step will be to increase the follow-up period and expand the sample size of the case to exclude its individual survivor effect.

There is currently no effective therapy to treat patients with SCA8, despite its symptomatic nature. Management of SCA8 is supplemented with systematic and rehabilitation therapy. Several studies support the effectiveness of synergistic physiotherapy in the management of SCA8 (Ashizawa et al., 2018). Because the primary functional impairment of the proband is spastic in nature, we used standard antispasmodic treatment strategies (Mugglestone et al., 2012). During the first stage of treatment in our hospital, which mainly consisted of physical therapy, the patient made negligible progress and her MAS scores were still high. According to expert consensus on the therapeutic application of botulinum toxin (Wan et al., 2018), botulinum toxin has a strong nerve blocking effect; thus, injection of botulinum toxin into spasmodic muscles can improve antagonistic muscle activity. In the second treatment stage, with the addition of botulinum toxin injection, the patient's lower limb flexor muscle tone reduced significantly, and lower limb MAS scores improved, but the mRS score was still poor. Oral baclofen is known to effectively alleviate the degree of spasm in children and improve the range of motion in joints (Huang et al., 2022). Baclofen can result in the hyperpolarization of anterior horn cells; as a result, muscle traction reflex and clonus are inhibited, producing skeletal muscle relaxation and, thus, relief from spasms (Ertzgaard et al., 2017). To effectively alleviate the spasms in the lower limbs of the patient, baclofen pumps were surgically implanted. The spasms of her lower limbs significantly improved, and both the mRS and MAS scores improved. The patient could now stand on her own and walk with flat feet with others' help. Potential treatments for SCA exist. Nucleotide-based gene silencing methods that target the first step in a pathogenic cascade are promising, not only for polyglutamine SCA but also for many other SCA types caused by toxic mutant proteins or RNA (Ashizawa et al., 2018). Presently, the Japanese Ministry of Health has approved the use of a thyrotropin-releasing hormone mimetic agent (taltirelin hydrate) for the treatment of SCA, but the use of this agent has not been approved in other countries (Ashizawa et al., 2018). With increasing understanding of the genetics and pathological mechanisms surrounding SCA8, promising therapeutic targets may potentially delay disease progression. A successful treatment for one or more SCA variants appears imminent (Buijsen et al., 2019).

## Conclusions

The clinical features, genetic analysis, and treatment of a family with an SCA8 lineage were reported in this paper. To our knowledge, till date, we are the first to report SCA8 affecting a Chinese family with an onset of spastic paraplegia in early childhood, where cerebellar ataxia is not the core symptom. Finally, we found that early, sequential, and comprehensive treatments can improve lower extremity motor function and the daily life of affected patients. This study provides valuable information regarding the expanding manifestations and effective symptomatic treatment of SCA8.

## Data availability statement

The original contributions presented in the study are included in the article/supplementary material, further inquiries can be directed to the corresponding author.

## Ethics statement

The studies involving human participants were reviewed and approved by the Ethics Committee of the Children's Hospital affiliated with Chongqing Medical University. Written informed consent to participate in this study was provided by the participants' legal guardian/next of kin. Written informed consent was obtained from the participant(s) for the publication of this case report.

## Author contributions

SC interpreted medical exome results and wrote the original manuscript draft. SL analyzed aggregated clinical data. YL and RS collected and reviewed the clinical data. WJ supervised and critically reviewed the study and revised the article. All authors reviewed the draft and approved manuscript submission for publication.
